# Five-Year Persistence of Vedolizumab in Crohn’s Disease: Results from a Real-World Cohort

**DOI:** 10.3390/jcm15041387

**Published:** 2026-02-10

**Authors:** Marc Harb, Vinciane Muls, Alice Hoyois, Jennifer Aoun

**Affiliations:** CHU Saint Pierre Brussels, Rue aux Laines 105, 1000 Brussels, Belgium; vinciane.muls@stpierre-bru.be (V.M.); alice.hoyois@stpierre-bru.be (A.H.); jennifer.aoun@stpierre-bru.be (J.A.)

**Keywords:** vedolizumab, Crohn disease, Crohn’s disease support tool

## Abstract

**Background**: Vedolizumab (VDZ) is an α4β7 anti-integrin monoclonal antibody effective in Crohn’s disease (CD). While its short- and mid-term efficacy is well established, real-world data on long-term outcomes beyond 3 years are scarce. Recent studies suggest a progressive decline in persistence rates after 2 to 3 years, with very limited data beyond this period. The primary objective of this study was to evaluate the 5-year persistence of VDZ. Secondary objectives were to describe clinical, biological, and endoscopic responses at 2 years among patients remaining on treatment, and to identify predictors of long-term persistence, including the baseline Clinical Decision Support Tool (CDST) score. **Methods**: We conducted a retrospective observational study which included 60 adult patients with CD treated with VDZ before April 2025. Collected baseline variables included age, sex, BMI, smoking status, disease duration and location, prior biologic exposure, and CDST score. Treatment persistence was evaluated at 5 years. Clinical, biological, and endoscopic responses were assessed at 2 years. A global response was then defined as the achievement of a clinically significant improvement, normalization or marked reduction in inflammatory biomarkers, and endoscopic improvement. Predictors of persistence were also analyzed. **Results**: The mean age of this cohort was 45.4 ± 15.2 years, mean disease duration was 12.7 ± 10.1 years, and mean CDST score was calculated at 20.6 ± 2.7. At 5 years, 23/41 patients (56.1%) remained on VDZ therapy. Persistence was significantly associated with male sex (65.2% vs. 27.8%), longer disease duration (215 vs. 106 months), absence of rheumatologic manifestations (13.0% vs. 44.4%), and clinico-biological response at 12 months (65.2% vs. 30.8%). At 24 months, a global response was observed in all patients persisting at 5 years compared to 22.2% of those who discontinued (*p* < 0.001). At 2 years, 39/51 patients (76.5%) remained on VDZ. Persistence was associated with longer disease duration (189 vs. 75 months), male sex (61.5% vs. 25.0%), and absence of isolated colonic disease (0% vs. 16.7%). A global response at 2 years was achieved by 89.7% of persistent patients compared with none of the non-persistent group (*p* < 0.001). The CDST, uniformly elevated in this cohort, did not discriminate between persistent and non-persistent patients, but reflected appropriate initial patient selection. **Conclusions**: This real-world study documents 5-year outcome data on VDZ persistence in Crohn’s disease, a duration infrequently studied, with 56% of patients maintaining treatment. Early response at 12 and 24 months emerged as a key determinant of long-term persistence, highlighting the value of assessing 2-year outcomes to identify durable responders. Although not discriminatory in this homogeneous cohort, the CDST score emphasizes the potential role of predictive tools in guiding personalized therapeutic strategies. These results contribute to defining the long-term role of VDZ in the management of CD.

## 1. Introduction

Vedolizumab (VDZ) is a recombinant humanized monoclonal antibody that selectively binds to the α4β7 integrin expressed on gut-specific lymphocytes [1].

VDZ has demonstrated significant efficacy in inducing and maintaining clinical remission in patients with Crohn’s Disease (CD), especially in those who have failed conventional therapies.

In Belgium, VDZ is recognized as a first-line treatment option for CD, which gives patients a distinct advantage in terms of early intervention and effective disease management. This is in contrast to many other countries where VDZ is not yet approved for first-line use and is typically considered only after failure of other biologic therapies. The opportunity to use VDZ as a first-line option in Belgium provides clinicians with greater therapeutic flexibility in managing the disease.

In real-world clinical practice, predicting therapeutic outcomes remains challenging, with a substantial number of patients with inflammatory bowel disease (IBD) experiencing either primary or secondary loss of response to biologics.

Additionally, the absence of well-defined strategies for therapeutic sequencing further complicates treatment choices. Therefore, identifying patients who are most likely to respond to biologic therapies, such as VDZ, prior to initiating treatment is crucial.

Given the growing demand for personalized treatment strategies to identify patients most likely to benefit from VDZ, Dulai et al. [2] developed and validated a scoring system in 2018 to predict which patients with Crohn’s disease are most likely to respond of VDZ therapy called the Clinical Decision Support Tool (CDST).

This score incorporates prior bowel surgery, prior anti-TNF therapy, prior fistulizing disease, baseline serum albumin and CRP as clinical variables in the equation. Based on the total score, patients are stratified into three prognostic categories: a high score (≥19) reflects a high likelihood of achieving clinical remission and maintaining treatment persistence; compared to those with a low score (≤12) who have a significantly reduced probability of response and long-term persistence. A score between 12 and 19 represents the intermediate score with moderate probability of clinical response [3].

However, the persistence of VDZ treatment over time, particularly at the 5-year mark, remains a critical area of study, as it may vary based on patient characteristics and prior treatment history.

For this purpose, we conducted a monocentric retrospective study including patients followed for Crohn’s disease and treated with VDZ at our institution. The primary objective was to examine the rate of VDZ persistence at 5 years in our studied population, as well as to describe the characteristics of these patients and to identify features associated with long-term treatment persistence.

The secondary objectives of this study were to describe the population of patients with treatment persistence at 2 years, evaluating their clinical, biological, and endoscopic responses. Finally, we sought to assess the association between the baseline CDST scores and treatment outcomes at two and five years.

## 2. Materials and Methods

This retrospective observational study included 60 patients (aged >18 years) with a confirmed diagnosis of CD who received VDZ therapy at CHU Saint-Pierre University Hospital in Brussels, before April 2025. Patients were identified through electronic medical records.

All patients received VDZ as monotherapy. Patients who received corticosteroids during the study period were excluded from the analysis.

Variables analyzed included age, sex, BMI, smoking status, disease duration, prior biologic exposure (bio-naive vs. bio-exposed), disease location, and CDST score.

Following this analysis, we assessed the persistence rate of VDZ at 5 years. We further described the characteristics of these patients to identify potential factors associated with long-term treatment persistence.

Treatment response at 12 months was defined as clinical improvement based on the Harvey–Bradshaw Index (HBI) and biological improvement based on C-reactive protein (CRP) and fecal calprotectin (FC).

Furthermore, at 24 months, treatment response was assessed through clinical activity, biological activity, and endoscopic parameters, the latter evaluated using the Simple Endoscopic Score for Crohn’s Disease (SES-CD).

Treatment persistence was defined as continuous VDZ use for five years without discontinuation due to lack of efficacy or adverse events. Interruptions for reasons unrelated to efficacy or safety, such as planned surgery, pregnancy, or loss to follow-up, were excluded. Patients who continued VDZ under these criteria for the full five-year period were considered responders.

Clinical response was defined as a reduction of at least 50% in the HBI score. Biological response was assessed by a decrease of 50% in CRP or fecal calprotectin compared with the preceding 12 months, and endoscopic response was defined as an improvement of at least 50% in the SES-CD score (Table 1).

At 12 months, an overall response was observed, defined by achievement of both clinical and biological endpoints. At 24 months, the overall response was documented in patients meeting at least two of the three predefined endpoints—clinical, biological, and endoscopic—indicating a sustained, multidimensional treatment effect.

## 3. Statistical Analysis

Statistical analyses were conducted using Stata version 18 (StataCorp, College Station, TX, USA). Continuous variables are presented as mean ± standard deviation (SD) or median with interquartile range (IQR). Categorical variables are summarized using frequencies and percentages. Student’s *t*-test, Mann–Whitney U test, χ^2^ test, or Fischer’s exact test were performed to compare demographic and clinical variables between groups. Univariate and multivariable regression analysis using the cox regression model was used to assess factors associated with treatment persistence. The results are presented as hazard ratios (HR) with 95% confidence intervals (CI). Kaplan–Meier survival curves were plotted and compared using the Log-rank test. All tests were two-sided and a *p*-value < 0.05 was considered statistically significant.

## 4. Results

### 4.1. Patients’ Demographics

This study included 60 patients with a mean age of 45.4 ± 15.2 years, comprising 31 men (51.7%) and 29 women (48.3%). The mean BMI was 26.8 ± 5.0 and 48.3% were smokers. The mean CDST score was 20.6 ± 2.7. The mean disease duration was 12.7 ± 10.1 years, with 53.3% having ileal disease, 41.7% ileo-colonic disease and 5% only colonic disease. Concerning biological treatment exposure, 61.67% of the population were bio-naive. These data summarize the key demographic and clinical characteristics of the study population (Table 2).

**Figure 1:**
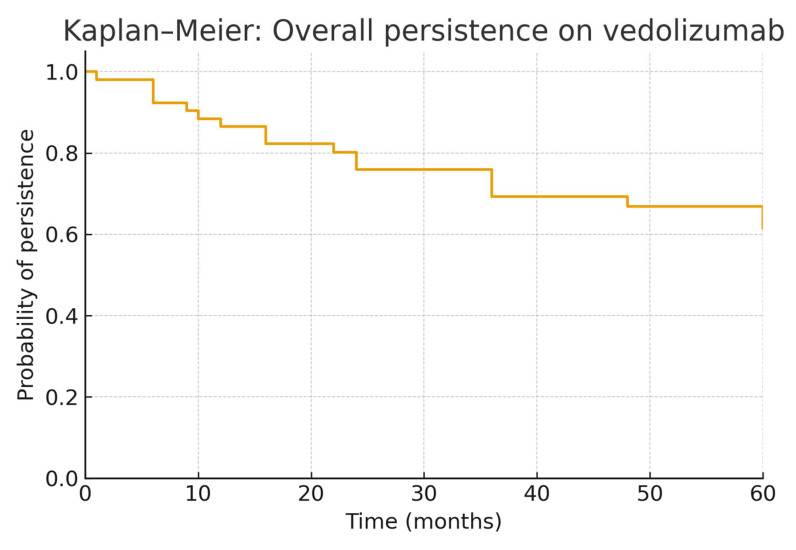
Kaplan–Meier: Overall persistence.

Among the 60 patients who initiated therapy, only 41 were eligible for analysis of 5-year treatment persistence, as the remaining 19 were still undergoing therapy at the time of evaluation and did not attain the 5-year interval. Of the 41 patients analyzed, 56.1% (*n* = 23) maintained treatment at 5 years (Table 3) (Figure 1).

### 4.2. Associated Factors for Treatment Persistence at 5 Years (Table 4)

Patients with sustained treatment were slightly older (49.4 ± 13.1 years) compared to those who discontinued (44.2 ± 17.6 years), although this difference was not statistically significant (*p* = 0.2354). The mean BMI was 25.5 ± 3.5 in the persistence group versus 27.5 ± 6.8 in the non-persistence group (*p* = 0.117). Notably, disease duration was significantly longer among persistent patients (215.3 ± 111.8 months) than non-persistent ones (105.8 ± 80.1 months; *p* = 0.001).

Persistence was significantly higher among male patients (65.2% vs. 27.8%, *p* = 0.074). No significant association was observed with smoking status, age at diagnosis (before or after 17 years), or disease location (ileal, colonic, ileocolonic).

Regarding prior biologic exposure, 43.5% of persistent patients are bio-naive, versus 66.7% of non-persistent patients, without statistical significance (*p* = 0.053). CDST scores were comparable between groups (20.5 ± 2.4 vs. 19.8 ± 2.3; *p* = 0.379).

Rheumatologic extra-intestinal manifestations were significantly associated with lower treatment persistence (13.0% vs. 44.4%; *p* = 0.028), and dermatologic extra-intestinal manifestations were also significantly associated with lower treatment persistence (*p* = 0.045). Ophthalmologic manifestations (*p* = 0.499) were not statistically significant.

Overall treatment response at 12 months was not significantly associated with 5-year persistence: 65.2% of persistent patients were in treatment response at 12 months compared to 30.8% of non-persistent patients (*p* = 0.134). A similar pattern was observed at 24 months, with all patients in the persistent group maintaining global response, versus only 22.2% in the non-persistent group (*p* < 0.001).

**Table 4:** Factors associated with VDZ treatment persistence at 5 years.

Factors	Persistent (*n* = 23)	Non-Persistent (*n* = 18)	Hazard Ratio [95% CI]	*p*-Value
Age, mean ± SD (years)	49.4 ± 13.1	44.2 ± 17.6	1.02 [0.98–1.08]	0.2354
Early CD * (N)	10	9	0.78 [0.32–1.94]	0.605
BMI, mean ± SD (kg/m^2^)	25.5 ± 3.5	27.5 ± 6.8	0.926 [0.84–1.01]	0.117
Smokers	52.2%	44.4%	1.20 [0.48–2.99]	0.687
Disease duration, mean ± SD (months)	215.3 ± 111.8	105.8 ± 80.1	1.01 [1.00–1.01]	0.0011
Male sex (%)	65.2	27.8	2.35 [1.05–5.24]	0.074
Isolated ileal disease (%)	34.8%	50%	0.51 [0.20–1.28]	0.156
Isolated colonic disease (%)	0	11.1	0.29 [0.15–0.55]	0.001
Ileo-colonic disease	60.9%	38.9%	2.27 [0.91–5.64]	0.075
Prior biologic exposure (%)	56.5	66.7	2.45 [0.98–6.09]	0.053
CDST score, mean ± SD	20.5 ± 2.4	19.8 ± 2.3	1.06 [0.92–1.23]	0.379
Rheumatologic EIM (%)	13.0	44.4	0.37 [0.15–0.90]	0.028
Dermatologic EIM (%)	4.3%	22.2%	0.35 [0.13–0.97]	0.045
Ophthalmologic EIM (%)	4.3%	11.1%	0.69 [0.24–2]	0.499
Global Response at 12 months (%)	65.2	30.8	1.96 [0.95–4.01]	0.134
Global response at 24 months (%)	100	22.2	4.50 [1.90–10.68]	0.001

* Early CD defined as diagnosis at ≤17 years of age.

### 4.3. Associated Factors for Treatment Persistence at 2 Years (Table 5)

This analysis included patients evaluated at 24 months, distinguishing those who continued treatment (first group) from those who discontinued it prematurely (second group). Among the total of 51 patients, 39 (76.5%) belonged to the first group and 12 (23.5%) to the second group. The mean age tended to be higher in the first group (48.5 ± 12.9 years) compared to the second group (39.4 ± 18.4 years), but this difference was not statistically significant (*p* = 0.061). Body mass index (BMI) was similar between the groups (26.4 vs. 27.0, *p* = 0.191). However, disease duration was significantly longer in the first group (189 ± 127 months) compared to the second group (75 ± 50 months), with a *p*-value of 0.0059.

The CDST score did not differ significantly between the groups (20.4 vs. 20.2, *p* = 0.772). Regarding sex distribution, males were more frequent in the first group (61.5% vs. 25.0%), whereas females predominated in the second group (38.5% vs. 75.0%), *p* = 0.065. Smoking status and early disease onset, before the age of 17, did not statistically differ between groups.

In terms of disease location, significant differences were observed for colonic involvement (0% in the first group vs. 16.7% in the second group, *p* = 0.001) and ileocolic involvement (53.8% vs. 16.7%, *p* = 0.0492), while ileal involvement alone was not significantly different.

Prior exposure to anti-TNF therapy was more frequent in the first group (51.3% vs. 16.7%, *p* = 0.0392). Extra-intestinal manifestations such as rheumatologic, dermatologic, and ophthalmologic involvement did not show significant differences between groups.

Regarding treatment response, a higher proportion of non-responders at 12 months was observed in the treatment discontinuation group (71.4% vs. 30.8%, *p* = 0.046). At 24 months, all patients evaluated showed a clinical response. However, biological, endoscopic, and overall response statuses at 24 months revealed significantly more non-responders in the second group, with *p*-values of 0.012, 0.001, and <0.001, respectively.

**Table 5:** Factors associated with VDZ treatment persistence at 2 years.

Factors	Persistent (*n* = 23)	Non-Persistent (*n* = 18)	Hazard Ratio [95% CI]	*p*-Value
Age, mean ± SD (years)	48.5 ± 12.9	39.4 ± 18.4	1.05 [0.97–1.13]	0.191
Early CD *	17	7	0.622 [0.20–1.93]	0.413
BMI, mean ± SD (kg/m^2^)	26.4 ± 4.09	27.0± 7.65	0.96 [0.81–1.13]	0.631
Smokers	18	6	0.87 [0.28–2.65]	0.812
Disease duration, mean ± SD (months)	189 ± 127	75 ± 50	1.01 [1.00–1.02]	0.0059
Male sex (%)	61.5	25.0	2.74 [1.09–6.90]	0.065
Isolated colonic disease (%)	0	16.7	0.22 [0.12–0.42]	0.001
Isolated ileal disease (%)	43.6%	66.7%	0.41 [0.13–1.32]	0.137
Ileo-colonic disease	53.8	16.7	1.62 [1.00–21.27]	0.0492
Prior biologic exposure (%)	51.3	16.7	4.60 [1.07–19.60]	0.0392
CDST score, mean ± SD	20.4 ± 3.01	20.2 ± 1.72	1.02 [0.89–1.16]	0.772
Rheumatologic EIM (%)	17.9%	41.7%	0.39 [0.13–1.20]	0.102
Dermatologic EIM (%)	10.3%	16.7%	0.56 [0.11–2.73]	0.4775
Ophthalmologic EIM (%)	7.7%	0%	3.96 [0.15–67.12]	0.730
Global Response at 12 months (%)	69.2	28.6	2.50 [1.13–5.53]	0.046
Global response at 24 months (%)	89.7%	0%	33.78 [2.19–521.6]	0.023

* Early CD defined as diagnosis at ≤17 years of age.

## 5. Discussion

The management of Crohn’s disease has evolved significantly with the introduction of biologic therapies, yet predicting which patients will achieve long-term benefit remains a challenge. In our cohort, we investigated VDZ persistence over 2 and 5 years, identifying clinical and biochemical factors associated with persistent therapy, thereby contributing to the understanding of treatment durability in routine clinical practice. Our study provides important evidence on how early treatment response, patient characteristics, and predictive tools such as the CDST can inform clinical decisions and optimize long-term outcomes. In our cohort, all patients had high CDST scores at baseline, reflecting a population already considered likely to respond to VDZ. This explains why the CDST could not effectively differentiate between persisters and non-persisters, and why statistical comparisons were not significant. These findings highlight that, in populations with uniformly high baseline scores, the discriminatory power of the CDST is inherently limited.

The differences observed between factors associated with treatment persistence at 2 years and at 5 years suggest that mid-term and long-term persistence may rely on partially distinct determinants. At 2 years, treatment persistence appears to be mainly associated with disease phenotype and early treatment-related factors, including disease location, prior exposure to anti-TNF therapy, and early clinical, biological, and endoscopic response.

In contrast, factors associated with 5-year persistence seem to be less related to initial treatment response and more closely associated with longer-term disease characteristics, such as longer disease duration and the absence of extra-intestinal manifestations. These findings suggest that while early therapeutic response plays a major role in mid-term treatment continuation, other disease-related factors may contribute to persistence over the long term. These observations should be interpreted with caution given the limited sample size at long-term follow-up and the exploratory nature of the analyses.

To our knowledge, our study is the only real-world cohort in Crohn’s disease reporting persistence with VDZ up to 5 years, with more than half of patients (56.1%) remaining on treatment at this time point. Reported persistence rates range from approximately 50–65% at 1 year, but they typically decline markedly at 2–3 years, influenced by factors such as prior biologic exposure and disease severity. Among other studies with shorter follow-up duration, the GEMINI LTS post hoc analysis showed a persistence rate of approximately 41% at 54 months in Crohn’s disease patients [4]. Furthermore, real-world data from a recent meta-analysis reported persistence rates of up to 65% at 1 year and 55% at 2 years, but did not reach the 5-year interval [5]. Similarly, in the prospective OBSERV-IBD study, Amiot et al. reported VDZ persistence rates of 48.5% at 1 year, 31.4% at 2 years, and only 26.3% at 3 years in Crohn’s disease patients [6].

Importantly, our results showed that early clinical, biochemical, and endoscopic outcomes in the first 1 to 2 years were significantly associated with maintenance of VDZ therapy at 5 years, highlighting the critical role of initial therapeutic success in predicting durable response.

These findings are consistent with the GEMINI LTS trial [4], where week-12 responders had higher VDZ persistence at 54 months (42% vs. 35%, *p* = 0.001), and the FINVEDO study [7], which reported 12-month persistence of 84.9% in Crohn’s disease patients, with significant improvements in clinical remission and biochemical outcomes.

Our results extend these observations by demonstrating that early responders can maintain and even improve their outcomes over 2 and 5 years. This complements the findings from the Swedish SVEAH cohort [8], where a lower baseline Harvey–Bradshaw Index (HBI) was associated with higher likelihood of clinical remission at week 156 (OR 0.87; 95% CI 0.78–0.96), reinforcing the importance of baseline disease activity and early response.

Moreover, consistent with the Swedish study’s findings of significant decreases in inflammatory biomarkers, plasma C-reactive protein decreasing from 5 mg/L at baseline to 4 mg/L at 156 weeks (*p* = 0.01), and fecal calprotectin from 641 µg/g to 114 µg/g (*p* < 0.01), our study reinforces the prognostic value of integrating clinical and biological response to guide long-term management in Crohn’s disease.

While differences in study design, patient population, and treatment protocols limit direct comparisons, the high VDZ continuation and remission rates reported in the Swedish cohort substantiate the clinical relevance of early response markers, aligning well with our findings on predictors of sustained remission.

Overall, these results indicate that baseline patient characteristics, early clinical response, and endoscopic outcomes are critical determinants of long-term VDZ persistence. Patients who respond well early in treatment are more likely to sustain their therapeutic outcomes over time, providing valuable information for personalized long-term management in Crohn’s disease.

In our study, we observed that patients with longer disease duration were more likely to persist on VDZ at five years. This finding contrasts with data from the LOVE-CD [9] study, which showed that patients with early-stage Crohn’s disease (disease duration less than 2 years) achieved superior clinical and endoscopic outcomes. Specifically, 31% of patients in the early disease group reached clinical and endoscopic remission at week 26, compared with only 9% in the late disease group (*p* = 0.001).

The apparent discordance between our findings and LOVE-CD may be explained by several factors. First, differences in population and study design likely contribute: LOVE-CD was a prospective study focusing on short-term clinical and endoscopic outcomes, while our study reflects real-world persistence over five years, capturing long-term adherence rather than short-term response. Furthermore, patients with longer disease duration may have greater therapeutic experience, better understanding of their condition, and improved coping strategies, all of which can favor long-term persistence. These findings should be interpreted with caution, as they are cohort-specific and may be influenced by local prescribing practices, patient selection criteria, and other contextual factors. Such considerations underscore the need for careful extrapolation of our results to other populations.

We also observed that male sex was associated with greater treatment persistence. This observation contrasts with most large clinical trials and real-world cohorts, where sex has not been consistently identified as an independent predictor of persistence. While most large extension studies, including the GEMINI long-term safety trial, did not identify sex as an independent predictor [4], our finding may reflect cohort-specific characteristics, such as differences in adherence, health behavior, or disease management.

Also, in the Swedish prospective multicentric SVEAH [8] extension study, female sex was associated with lower rates of clinical remission at week 156 in univariate analysis (OR 0.46, 95% CI 0.23–0.94), but this association was no longer significant after multivariable adjustment (OR 0.65, 95% CI 0.28–1.50), indicating that sex is not an independent predictor of long-term outcomes. These findings suggest that while sex may influence treatment outcomes in certain analyses, it does not appear to be a consistent independent predictor of long-term VDZ persistence.

Cohort-specific characteristics, behavioral factors, or disease severity may account for the associations observed in some populations, highlighting the need for further research to explore potential gender-specific modifiers of treatment adherence and response.

We explored whether rheumatologic extra-intestinal manifestations represent an additional factor influencing 5-year VDZ treatment persistence. In our cohort, patients with such manifestations had significantly lower persistence, with only 13.0% remaining on therapy compared to 44.4% of those without. These findings align with published data on the impact of VDZ on rheumatologic manifestations in Crohn’s disease. Huseynzada et al. (2023) [10] reported that, among 29 patients with Crohn’s disease, VDZ had no significant effect on spondyloarthritis symptoms, with some patients developing new manifestations or experiencing exacerbation of pre-existing symptoms. Similarly, Varkas et al. (2017) [11] reported that VDZ could induce or worsen arthritis and/or sacroiliitis in patients with Crohn’s disease. Together, these data suggest that while VDZ is effective in controlling intestinal inflammation, its impact on rheumatologic manifestations is limited, and such manifestations may contribute to reduced long-term treatment persistence.

Prior exposure to anti-TNF agents may influence the long-term use of VDZ, with variable findings reported in the literature. The POLONEZ II study (2024) [12] followed a cohort of Crohn’s disease patients for 54 weeks in a real-world setting to evaluate VDZ’s effectiveness and persistence. This study revealed no significant differences were observed between biologic-naïve patients and those previously exposed to biologics.

On the other hand, the Belgian ECCO Registry [13] showed that clinical remission was achieved in 67% of CD patients and was higher for patients without prior anti-TNF therapy (87%) than for those previously exposed to anti-TNF agents (70%). This emphasizes the role of prior biologic exposure in predicting long-term treatment adherence with VDZ.

In contrast, in our cohort, although the difference did not reach statistical significance, we observed a trend toward better treatment persistence in patients previously exposed to biologics compared with biologic-naïve patients (56.5% vs. 33.3%, *p* = 0.139), a finding that might be related to the relatively small sample size.

With the growing use of biologic agents for IBD over the past decade, clinical prediction models have become valuable tools to help guide therapeutic decisions.

The 5-variable VDZ CDST, originally developed from the phase 3 GEMINI 2 trial and later validated in the real-world VICTORY consortium, has been shown to identify Crohn’s disease patients most likely to achieve a clinical response after 26 weeks of VDZ therapy.

In our study, CDST also appeared to be a relevant predictor of treatment persistence, as all patients had baseline scores above 19, whether they persisted or discontinued VDZ at 2 and 5 years. This suggests that while a high CDST score may indicate an increased likelihood of initial response, it does not necessarily distinguish long-term persisters in our cohort.

This limitation is likely related to the retrospective design, since most patients selected for VDZ already met the threshold score, thereby reducing the discriminatory capacity of the tool. In addition, the relatively small sample size may have limited the ability to detect subtle differences.

Finally, treatment persistence is shaped not only by baseline clinical features captured by CDST, but also by subsequent disease course, treatment tolerance, and patient preferences, which are not fully accounted for by the score.

Several studies have also explored the correlation between Clinical Decision Support Tool (CDST) scores and VDZ treatment persistence. Alric et al. (2022) [14] found that the CDST score predicts clinical remission and steroid-free clinical remission at week 48 for VDZ but not for ustekinumab in CD patient’s refractory or intolerant to anti-TNF.

In addition, in the Korean KASID [15] multicenter study, which included 71 patients with Crohn’s disease treated with VDZ, the clinical decision support tool showed only a modest ability to predict which patients would achieve steroid-free clinical remission at 26 weeks. Patients with a higher probability score did have better outcomes than those with lower or intermediate scores, but overall, the tool was only moderately effective in distinguishing responders from non-responders. This contrasts with our findings, where patients in our cohort had a relatively high average score and demonstrated markedly higher long-term treatment continuation over five years. These differences may reflect variations in patient populations and highlight the need for a prospective study to confirm whether a higher baseline score can reliably predict long-term persistence with VDZ.

Our study has several limitations. The cohort was relatively small (*n* = 60), which may have limited the statistical power to detect subtle differences. The retrospective design may have introduced selection and information biases. Not all patients in the cohort had reached the full 5-year follow-up at the time of data collection. Consequently, nineteen patients were excluded from the analysis of long-term vedolizumab persistence. This exclusion may introduce attrition bias, as patients with shorter follow-up might have different outcomes compared with those who persisted on therapy. Therefore, the persistence rates reported in this study could be slightly overestimated. This limitation should be considered when interpreting the long-term durability of VDZ treatment in this real-world cohort. Regarding the borderline results observed, the analyses of factors associated with treatment persistence were exploratory in nature. These observations should be interpreted with caution and require validation in larger, independent cohorts to confirm their potential predictive value. In addition, all patients had high baseline CDST scores, limiting the ability to compare outcomes between low and high scores and to fully evaluate the correlation between CDST and treatment persistence. Furthermore, this study was conducted in a single country and included patients already selected for VDZ, which may reduce the generalizability of our findings. Differences in clinical management and follow-up between physicians could also influence persistence outcomes.

## 6. Conclusions

This 5-year real-world retrospective cohort study demonstrated a notably high rate of long-term treatment persistence, with 56.1% of patients maintaining clinical response at 5 years. Such an extended follow-up period adds valuable insight into the durability of therapeutic benefit in Crohn’s disease. The inclusion of endoscopic response at 24 months as a predictive factor is a key strength of our study, reinforcing the importance of objective disease activity assessment beyond clinical symptoms alone. Although the CDST score was initially developed to predict response to VDZ, it did not significantly differ between patients with and without long-term persistence in our cohort. Several independent predictive factors of sustained response were identified, including male sex, longer disease duration, absence of rheumatologic manifestations, and overall clinical response at 24 months. These findings contribute to refining patient stratification strategies for long-term management. In particular, longer disease duration, prior biologic exposure, and early clinical response appear to be key predictors of durable persistence with VDZ.

## Figures and Tables

**Table 1:** Treatment response at 12 and 24 months.

Timepoint	Clinical Response (%)	Biological Response (%)	Endoscopic Response (%)
12 months	≥50% reduction in HBI	≥50% reduction in CRP or FC	Not assessed
24 months	≥50% reduction in HBI	≥50% reduction in CRP or FC	≥50% reduction in the SES-CD

HBI: Harvey–Bradshaw Index, CRP: C-reactive protein, FC: Fecal calprotectin, SES-CD: Simple Endoscopic Score for Crohn’s Disease.

**Table 2:** Baseline characteristics of the study population.

Characteristics	*N* = 60
Age, mean ± SD (years)	45.4 ± 15.2
Male/female (%)	31 (51.7)/29 (48.3)
BMI, mean ± SD (kg/m^2^)	26.8 ± 5.0
Smokers (%)	48.3%
Disease duration, mean ± SD (years)	12.7 ± 10.1
Bio-naïve patients (%)	61.67%
Ileal disease/ileo-colonic disease/isolated colonic (%)	53.3%/41.7%/5%
CDST score, mean ± SD	20.6 ± 2.7

BMI: Body Mass Index, CDST: Clinical Decision Support Tool.

**Table 3:** VDZ treatment persistence at 2 and 5 years.

Time Point	Persistence Rate % (*N*)	Number of Patients, *N*
2 years	76.5% (39)	51
5 years	56.1% (23)	41

## Data Availability

The data analysed in this study are derived from existing patient records held by the authors’ institution. Due to privacy and confidentiality restrictions, these data are not publicly available, but aggregated data can be provided upon reasonable request.
